# Prevalence of and Some Factors Relating with Unwanted Pregnancy, in Ahwaz City, Iran, 2010

**DOI:** 10.5402/2011/523430

**Published:** 2011-10-27

**Authors:** M. Najafian, K. B. Karami, M. Cheraghi, R. Mohammad Jafari

**Affiliations:** ^1^Department of Obstetrics & Gynecology, Medical School, Ahwaz Jundishapur University of Medical Sciences, Ahwaz 61357-15751, Iran; ^2^Department of Public Health, Ahwaz Jundishapur University of Medical Sciences, Ahwaz 61357-15751, Iran

## Abstract

We *aimed* to find the prevalence and some factors relating with unwanted pregnancy. *Methods*. It was a cross-sectional study on 400 randomly pregnant women, who were referring to different health centers in Ahwaz city during 2010. Data was conducted based on questionnaire, and all the analysis was performed using SPSS (version 17) statistical analysis software. *Results*. The prevalence of unwanted pregnancy was 26%. The percentage of unwanted pregnancy in ages more than 35 years was approximately three times more than the intended pregnancy. There were significant relationship between unwanted pregnancy and some variables such as age, number of pregnancy, number of childbirth, education status, economic status, husband's occupation, and the relationship with the spouse and contraceptive methods (*P* < 0.0001). *Conclusion*. The prevalence of unwanted pregnancy was high. To prevent unwanted pregnancy using consultation services before planning to be pregnancy, it is necessary to identify the factors relating with unwanted pregnancy.

## 1. Introduction

Attempts to confine reproduction refer to a distant time. Only those contraceptive methods are new in which synthetic steroid is used [1]. Regarding the current status of growth, the world population is doubled every 54 years; however, in poor countries, population is doubled in less than 20 years. To complete and preserve personal health during pregnancy, the optimal use of contraceptive methods is effective, and planning is necessary before pregnancy [2]. The rate of pregnancy in women with a high potency for fertility who use no contraceptive method is 90% during one year. A proved view is that the prevention is less dangerous than the pregnancy itself [3]. Therefore, conscious and accurate decision making for pregnancy and precise care play an important role in decreasing the maternal mortality. Induced abortion that can be the important complication followed by unwanted pregnancy play a significant role in the incidence of infection, fever, risk of the next premature delivery, low birth weight, and infertility [1, 4]. Pregnancy is a temporary crisis that creates deep mental, physical, and behavioral changes in a woman. Conscious and accurate decision making for pregnancy, continuation, and precise care play an important role in declining the maternal mortality. According to the studies by World Health Organization, close to one-third of the pregnancies in the third world countries are unwanted [5]. This kind of pregnancy increases the incidence chance of complication and maternal mortality [2]. Considering the fact that induced abortion may create some significant complications such as infection, septic shock, fever, risk of the next premature delivery, low birth weight, and infertility, it can also cause some anatomic complications resulting from the surgery such as uterus, bladder, and intestinal rupture [1, 4]. Planning for pregnancy can help the safety of childbirth, and unplanned pregnancies increase the maternal mortality [2]. Despite the attempts by the authors in health centers, some unwanted pregnancies occur that can danger the women. Therefore, we decided to evaluate some factors related to unwanted pregnancy in women referred to health centers.

## 2. Methods

It was a cross-sectional study on 400 randomly pregnant women, who were referred to several clinics and health center in Ahwaz city during 2010.

Data was collected through interview and filling up a designed questionnaire containing demographic characteristics, fertility, and so forth. All the analysis was performed using SPSS (version 17) software. Descriptive and chi-square test analysis had seen performed to this study.

## 3. Results

**Figure 1:**
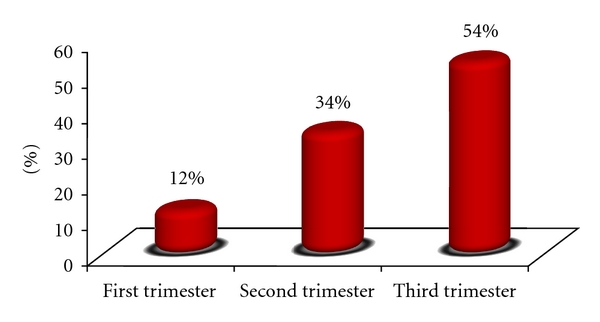
Prevalence of unwanted pregnancy in different trimester.

The prevalence rate of unwanted pregnancy was 26%. The mean age of women with unwanted pregnancy was 27.5 ± 5.7 years, and in women with intended pregnancy was 24.6 ± 4.5 years. The percentage of the older women (≥35 years) in unwanted pregnancy was 3 times of intended pregnancy which was statistically significant. Most of pregnant women lived in urban area (70.1%), and the percentage of rural women who intended pregnancy was higher (35.6%). The educational level in the subjects was 36% in secondary school and 44% in high school. 37% of subjects with unwanted pregnancy were illiterate and primary school (Table 1). The findings have shown that most of pregnant women were housewives (82%), and their husbands had self-employment (40%), and 7.1% of subjects with unwanted pregnancy had unemployed husband. Low economic status was higher in unwanted pregnancy. Half of the women with unwanted pregnancy had low economic status, while, in women with intended pregnancy, this rate was only 20%. According to the interviews, good relationship with the husband in women with unwanted pregnancy was lower (66%). Regarding fertility characteristics, the findings showed that more than half of the women with unwanted pregnancy were in the third trimester 54%, (Figure 1). 80% of unwanted pregnancy had more than two times pregnancies, but the percentage in intended pregnancy was 38%. This study showed that more than half of the pregnant women had used one of the contraceptive methods before the recent pregnancy, and 30% had used natural (interrupted) methods. The most percentage of women with unwanted pregnancy used unreliable methods like interrupted method (59.1%). The incidence of pregnancy followed by the consumption of contraceptive pills in women unwanted pregnancy was 16%. This study has shown that 26% of women who wish to become pregnant had more knowledge and performance about pregnancy health 26%, and the low levels of knowledge and performance were more observed in unwanted pregnancy.

## 4. Discussion

The incidence of unwanted pregnancy is different in the world, but it has the same undesired outcomes. Our study has shown that prevalence of unwanted pregnancy was 26%, and similar conducted studies indicated that the incidence rate of unplanned pregnancy was 25%, 30%, 43%, and 52% [6–9]. In this study, relative decrease in the incidence of unwanted pregnancy in our country in comparison with the other countries may be due to the hopeful success of health centers. Considering the point that intended pregnancy which results in a healthy childbirth from a healthy mother as an aim of midwifery science [10, 11], pregnancy must be occurred based on the accurate and conscious decision and according to the physical, mental, economic, social, and cultural status [2, 3]. Alenova in a study after evaluating the activities of consultation clinics guiding women for that the prevention of pregnancy states that prevention of unwanted pregnancy is more necessary in aged women, and it becomes more vital with the increase of age [12]. Mohammadloo in his study declares that more than half of the unwanted pregnancies occur in women more than 30 years [13]. One of the causes of not intending pregnancy is the age of more than 35 years that has been noted in other studies too [6, 8, 13, 14]. In the present study, low education has a close relationship with increased incidence of unwanted pregnancy, and, in a study by Bennett et al., this rate is doubled in low education [15]. This indicates that individuals with low level of education need more consultation services. 

Most of the women have the ability to become pregnant for at least three decades in their life, but most of men are potentially fertile all over their life [13]. In our study, the rate of unwanted pregnancy was higher in individuals with more number of children. Also, in similar studies, the increased prevalence of unwanted pregnancy is observed with an increase in the number of children, from 7.9% in childless women to 92.8% in women with 4 children or more [10]. In our study, unwanted pregnancy was more observed in employee women, and some other studies have achieved this result too [6]. Low income, poverty, unemployed husband, and inappropriate job play significant role in the incidence of unplanned pregnancy [7, 15–19]. A study in Zimbabwe has shown that women with unemployed husbands were more exposed to the unwanted pregnancy [20]. Some studies in Africa [7] and New York [17] indicated that unwanted pregnancy more occurred in poor, low income, and homeless women, which necessitates more concern about the poor women. Other studies in Thailand [21] and USA [22] have found that one of the related factors with unwanted pregnancy is the relationship with the spouse; also they showed that good relationship with the spouse in women with unwanted pregnancy was less than intended cases. A study from London has found that planned pregnancy was more observed in couple with more strengthened union in marriage [7]. It seems that couples' relationship is an important and positive factor which affects the increase of their cooperation in regarding the fertility health [23]. It was indicated in this study that, in 20% of women with unwanted pregnancy, no contraceptive method was used, and more than half of them used unreliable methods of which failure was more observed in unwanted pregnancies. A study in Egypt states that 47% of pregnant women with unplanned pregnancy do not use adequate prevention, and 28.8% encounter with the failure of their contraceptive method [6]. In a study in China, it has been indicated that the failure of the contraceptive method has been the main cause of unwanted pregnancy [24]. It seems that of the most important educational needs and the most effective attempts to prevent unwanted pregnancy are creating motivations in families, providing necessary facilities, and helping them in selection and accurate use of different methods by holding educational classes in health centers. Therefore, introducing accurate information about the contraceptive methods acquires a profound understanding, and using this information is very important in planning for the pregnancy [25]. Considering the lack of safe methods, educational programs, introducing adequate contraceptive options, and consultation before pregnancy are effective strategies for decreasing unwanted pregnancy and its outcomes [24]. 76% of world population live in developing countries, 85% of births, 95% of neonatal mortality, and 99% of maternal mortality occur in these countries [25]. So, regulating reproduction and adequate planning for pregnancy is significant in these countries. 

In conclusion, more attempts must be taken to decrease complication of pregnancies such as unwanted cases. In the recent years, it has been proved that, in addition, the population control and educational program for contraceptive methods are important and necessary in preventing unwanted pregnancy. Because in Iran abortion is illegal consider in the Muslim religious, unwanted pregnant women cannot do abortion except if physician has diagnosis that mother has complication to continue of her pregnancy or there is intrauterine growth retardation in the first trimester. 

## Figures and Tables

**Table 1:** level of education in intended pregnancy as well as unwanted pregnancy.

	Intended pregnancy	Unwanted pregnancy
Primary education or illiterate	20%	37%
Secondary education	36%	28%
High school	44%	35%
